# Performance of Artificial Intelligence Chatbots on Ultrasound Examinations: Cross-Sectional Comparative Analysis

**DOI:** 10.2196/63924

**Published:** 2025-01-09

**Authors:** Yong Zhang, Xiao Lu, Yan Luo, Ying Zhu, Wenwu Ling

**Affiliations:** 1 Department of Medical Ultrasound, West China Hospital of Sichuan University, 37 Guoxue Alley, Chengdu, 610041, China, 86 18980605569; 2 Department of Thoracic Surgery, West China Hospital of Sichuan University, Chengdu, China

**Keywords:** chatbots, ChatGPT, ERNIE Bot, performance, accuracy rates, ultrasound, language, examination

## Abstract

**Background:**

Artificial intelligence chatbots are being increasingly used for medical inquiries, particularly in the field of ultrasound medicine. However, their performance varies and is influenced by factors such as language, question type, and topic.

**Objective:**

This study aimed to evaluate the performance of ChatGPT and ERNIE Bot in answering ultrasound-related medical examination questions, providing insights for users and developers.

**Methods:**

We curated 554 questions from ultrasound medicine examinations, covering various question types and topics. The questions were posed in both English and Chinese. Objective questions were scored based on accuracy rates, whereas subjective questions were rated by 5 experienced doctors using a Likert scale. The data were analyzed in Excel.

**Results:**

Of the 554 questions included in this study, single-choice questions comprised the largest share (354/554, 64%), followed by short answers (69/554, 12%) and noun explanations (63/554, 11%). The accuracy rates for objective questions ranged from 8.33% to 80%, with true or false questions scoring highest. Subjective questions received acceptability rates ranging from 47.62% to 75.36%. ERNIE Bot was superior to ChatGPT in many aspects (*P*<.05). Both models showed a performance decline in English, but ERNIE Bot’s decline was less significant. The models performed better in terms of basic knowledge, ultrasound methods, and diseases than in terms of ultrasound signs and diagnosis.

**Conclusions:**

Chatbots can provide valuable ultrasound-related answers, but performance differs by model and is influenced by language, question type, and topic. In general, ERNIE Bot outperforms ChatGPT. Users and developers should understand model performance characteristics and select appropriate models for different questions and languages to optimize chatbot use.

## Introduction

With the rapid development of artificial intelligence (AI) technology, deep learning models are being increasingly and widely used in various fields, especially in natural language processing and computer vision [1,2]. In the field of natural language processing, several large pretrained models, such as OpenAI’s ChatGPT and Baidu’s ERNIE Bot [3,4], have demonstrated strong text generation and understanding capabilities. These models acquire rich semantic knowledge and language patterns through pretraining on large-scale corpora, which enables them to handle various complex natural language tasks [5,6]. In recent years, researchers have explored new algorithms and frameworks to optimize the performance of models and improve their accuracy and efficiency in handling complex tasks. In these studies, model selection, training, evaluation, and performance in practical applications have become the focus of research [7,8]. The proportion of medical health–related knowledge obtained through the internet is large, and chatbots are also used to answer various medical questions [9,10]. Researchers have performed many evaluations and studies on chatbots to answer medical questions, including ophthalmology, pediatric, urology, dentistry, and other professional directions, involving myopia, cirrhosis, hypertension, obesity, and other diseases and medical examination questions [11,12].

With the rapid development of ultrasound medicine, the demand for ultrasound examination is increasing, and the teaching and popularization of ultrasound is limited. An increasing number of junior ultrasound doctors, students, and patients have begun to use chatbots to obtain ultrasound-related consultation and answers. However, current research shows that chatbot performance is uneven; in some areas or tasks, chatbot performance can reach more than 90% of the accuracy rate or satisfaction, and chatbot performance can even exceed that of some doctors; however, in some tasks, the answer provided is not valid or even wrong [13,14]. There are performance differences among different models, which are also affected by many factors, such as language, question type, and topic [15]. An in-depth understanding of how models perform in various domains and under various conditions is necessary and valuable for both users and developers [16,17].

ChatGPT is a large-scale language model based on the transformer architecture developed by OpenAI, an American AI research laboratory. It simulates the process of human language generation and understanding through deep learning technology and adopts an autoregressive language modeling method to predict the next word or phrase in the text sequence to generate coherent text [18,19]. ChatGPT training data are derived from massive amounts of text data on the internet, including news reports, academic articles, and social media content. After the data are cleaned and labeled, the data are used to train the parameters of the model so that it can capture the complex patterns and semantic relationships of natural language [20,21]. ChatGPT’s powerful language generation and context understanding capabilities enable it to automatically generate relevant and coherent responses based on the input text content, enabling natural interaction with human users and completing multiple linguistic tasks, such as questions and answers, text summaries, and sentiment analysis [6,20,22].

ERNIE Bot is an intelligent question-and-answer system based on deep learning technology created by China’s Baidu. Its basic principle is to analyze and understand the questions raised by users by natural language processing technology, convert the questions of users into a form that computers can understand, and extract relevant information from massive amounts of data [23,24]. Natural language generation models are then used to transform the extracted information into a form that humans can understand, generate answers, and return them to the user. ERNIE Bot has a large amount of data, including trillions of web data, billions of search data and image data, billions of daily voice call data, and 550 billion facts in the knowledge graph [25]. ERNIE Bot uses advanced natural language processing algorithms to accurately understand user questions and provide precise answers. Furthermore, it supports personalized customization according to different scenarios and needs and can continue to learn and accumulate knowledge to improve intelligence.

The purpose of this cross-sectional study is (1) to evaluate and compare the performance of ChatGPT and ERNIE Bot in answering questions in ultrasound examination papers; (2) to comprehensively analyze and compare the performance differences of the models in different question types, topics, and input language environments; and (3) to explore the reasons for these differences. This study is expected to provide insight for chatbot users and developers so that we can better understand the performance of various models in different fields. For more complex medical problems and fields, we can attempt to combine the advantages of multiple models to make an objective comprehensive judgment and consider the results. Chatbots can provide better services while constantly improving their own performance.

## Methods

### Question Curation

**Figure 1.**
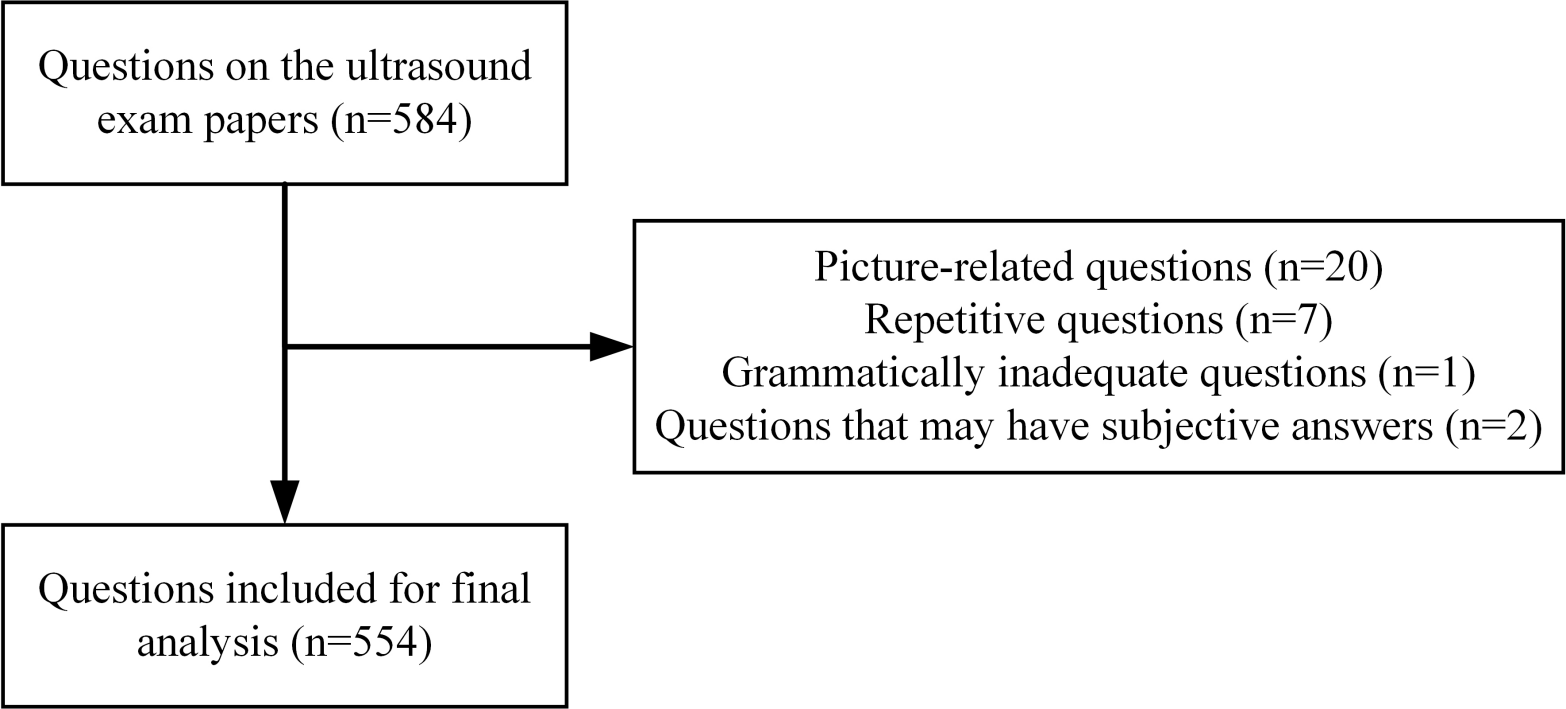
Flowchart of the questions included in the study.

We used questions from ultrasound examination papers as a data set. The questions, from the West China Clinical College of Medicine of Sichuan University, covered basic knowledge of ultrasound medicine, the digestive system, the urinary system, superficial organs, blood vessels, and the heart. The question types included single-choice, multiple-choice, true or false questions; noun explanations; and short answers. With a total of 584 questions, we excluded picture-related questions, repetitive questions, questions with poor grammar, and questions with subjective answers from this cross-sectional study. Finally, a total of 554 questions were included, and the flowchart of the questions suitable for inclusion in the study is shown in Figure 1.

### Response Generation and Grading

Each question was entered into GPT-3.5 Turbo (GPT-3.5) and ERNIE Bot-3.5 (Bot-3.5) in both English and Chinese, and the responses were recorded. For each input question, the background definition of the question, and the explanation of the question type are given, such as the noun explanation of the undergraduate superficial organ ultrasound examination. An example of the questioning methods for different types of questions is shown in Textbox 1. The subjective questions in this cross-sectional study included noun interpretation questions and short answer questions. All the subjective questions were scored by 5 experts in the field of ultrasound medicine who are fluent in both Chinese and English and have more than 20 years of experience in the fields of ultrasound diagnosis and ultrasound teaching. The experts rated the comprehensive quality of the responses in both languages on a Likert scale [26-28], which is an effective, objective, and fair evaluation method for quantifying and grading answers. The evaluation of subjective responses in this study includes completeness, logical clarity, accuracy, depth and breadth, creativity, and so on. A higher score indicates higher quality (1 point: very poor, the answer content seriously deviates from the requirements of the question, the logic is confused, and there is basically no correct content; 2 points: poor, part of the answer deviates from the requirements of the question, contains some correct content, but the logic is not clear enough, and there are some mistakes or omissions; 3 points: acceptable, the answers basically meet the requirements of the question, the content is relatively complete, the logic is relatively clear, but some details or explanations are not accurate or in depth; 4 points: good, the answer fully meets the requirements of the question, and the content is accurate, complete, and logical; and 5 points: very good, the answers fully meet the requirements of the question, the content is accurate and in depth, comprehensive, logical and rigorous, and even new insights or solutions are proposed). The experiments were completed in October 2024.

Textbox 1. **Examples of how to ask questions, including the content of the question, background, and question type description.**

**Example A**

The following is a single-choice question for the ultrasound examination. Please select the most appropriate one from the options given.

- Which of the following is true about ultrasound?

-  Ultrasound is an electromagnetic wave with a strong penetrating force.
-  The commonly used frequency range of medical ultrasound is 2.5-12 MHz.
-  Ultrasound wave is a wave with a frequency greater than 2000 Hz.
-  The form of ultrasound wave propagation in the human body is mainly shear wave.
-  Ultrasound is not easily affected by gas and bone.

**Example B**

The following is a multiple-choice question for the superficial organ ultrasound examination. Please select two or more of the correct answers from the options given.

- To which layers is the mammary gland subdivided by the fascia layer?

-  Skin layer
-  Subcutaneous fat layer
-  Glandular (parenchymal) layer
-  Fat layer in the retromammary space
-  Chest wall layer

**Example C**

The following is a judgment question for the ultrasound imaging examination. Please judge whether the following description is correct or not.

- An infective endocarditis patient can definitely detect growth on ultrasound.

**Example D**

The following is a noun explanation question for the superficial organ ultrasound examination. Please make an appropriate explanation of the following nouns.

- Acoustic impedance

**Example E**

The following is a short answer question for the ultrasound examination.

- Please briefly describe the common etiology and typical ultrasound features of cirrhosis.

### Statistical Analysis

For statistical analysis purposes, all the questions were grouped into categories: basic knowledge, disease and etiology, ultrasound examination, ultrasound diagnosis, ultrasound signs, case analysis, etc. We used Microsoft Excel to conduct statistical analyses (version 2019; Microsoft Corporation).

### Ethical Considerations

Ethics approval was not required since the research did not involve human subjects or animals.

## Results

All the questions included in this cross-sectional study are from real ultrasound examination papers, and the proportions of question types and categories are highly representative. The types and categories of all the questions included in this study are shown in Tables 1 and 2. Among the 554 eligible questions included in this study, according to the classification of question types, single-choice answers accounted for the highest proportion (354/554, 64%), followed by short-choice answers (69/554, 12%) and noun explanations (63/554, 11%), and the rest were multiple-choice and true or false questions. According to the classification of topics, the greatest proportion of topics were basic knowledge (181/554, 33%), followed by disease and etiology (106/554, 19%) and ultrasound signs (96/554, 17%), and the rest were ultrasound examination, ultrasound diagnosis, and ultrasound case analysis.

**Table 1.** Questions types and categories included in this study.

Categories	Basics, n	Examinations, n	Diagnosis, n	Cases, n	Disease, n	Signs, n	Total, n
Single choice	124	49	41	26	63	51	354
Multiple choice	13	8	6	—^a^	12	9	48
True or false	10	7	3	—	—	—	20
Noun explanation	21	8	3	—	24	7	63
Short answer	13	10	10	—	7	29	69
Total	181	82	63	26	106	96	554

a Not applicable.

**Table 2.** Study results for each question category, stratified by question style (single-choice, multiple-choice, and true or false questions), language (English and Chinese), and artificial intelligence model (GPT-3.5 Turbo [GPT-3.5] and ERNIE Bot-3.5 [Bot-3.5]).

Categories	Model	Basics, %	Examination, %	Diagnosis, %	Cases, %	Disease, %	Signs, %	Total, %
Single choice (English)	GPT-3.5	58.87	59.18	58.54	57.69	50.79	58.82	57.34
Correct (%)	Bot-3.5	57.26	61.22	58.54	61.54	63.49	60.78	59.89
Single choice (Chinese)	GPT-3.5	60.48	61.22	63.41	57.69	53.97	60.78	59.6
Correct (%)	Bot-3.5	58.06	65.31	56.1	65.38	68.25	70.59	62.99
Multiple choice (English)	GPT-3.5	7.69	0	16.67	—^a^	8.33	11.11	8.33
Correct (%)	Bot-3.5	46.15	12.5	16.67	—	25.0	66.67	35.42
Multiple choice (Chinese)	GPT-3.5	7.69	0	16.67	—	16.67	11.11	10.42
Correct (%)	Bot-3.5	53.85	12.5	16.67	—	33.33	66.67	39.58
True or false (English)	GPT-3.5	50	57.14	100	—	—	—	60
Correct (%)	Bot-3.5	60	85.71	100	—	—	—	75
True or false (Chinese)	GPT-3.5	50	71.43	100	—	—	—	65
Correct (%)	Bot-3.5	70	85.71	100	—	—	—	80

a Not applicable.

We collected the accuracy rate results of the 2 AI models, GPT-3.5 and Bot-3.5, for single-choice, multiple-choice, and true or false questions in English and Chinese. When the models were asked questions in Chinese (the original language of the test paper), the overall accuracy rate was as follows (GPT-3.5 vs Bot-3.5): single-choice (59.6% vs 62.99%), multiple-choice (10.42% vs 39.58%), and true or false questions (65% vs 80%). When the test paper was translated into English for questioning, the overall accuracy rates were as follows (GPT-3.5 vs Bot-3.5): single-choice (57.34% vs 59.89%), multiple-choice (8.33% vs 35.42%), and true or false questions (60% vs 75%). All translations are performed manually by experts who are proficient in English. It can be clearly seen that Bot-3.5 is superior to GPT-3.5 in all question types and languages. Furthermore, we calculated classified statistics according to the accuracy rates of different categories of questions, and the statistical results are shown in Table 2.

**Figure 2.**
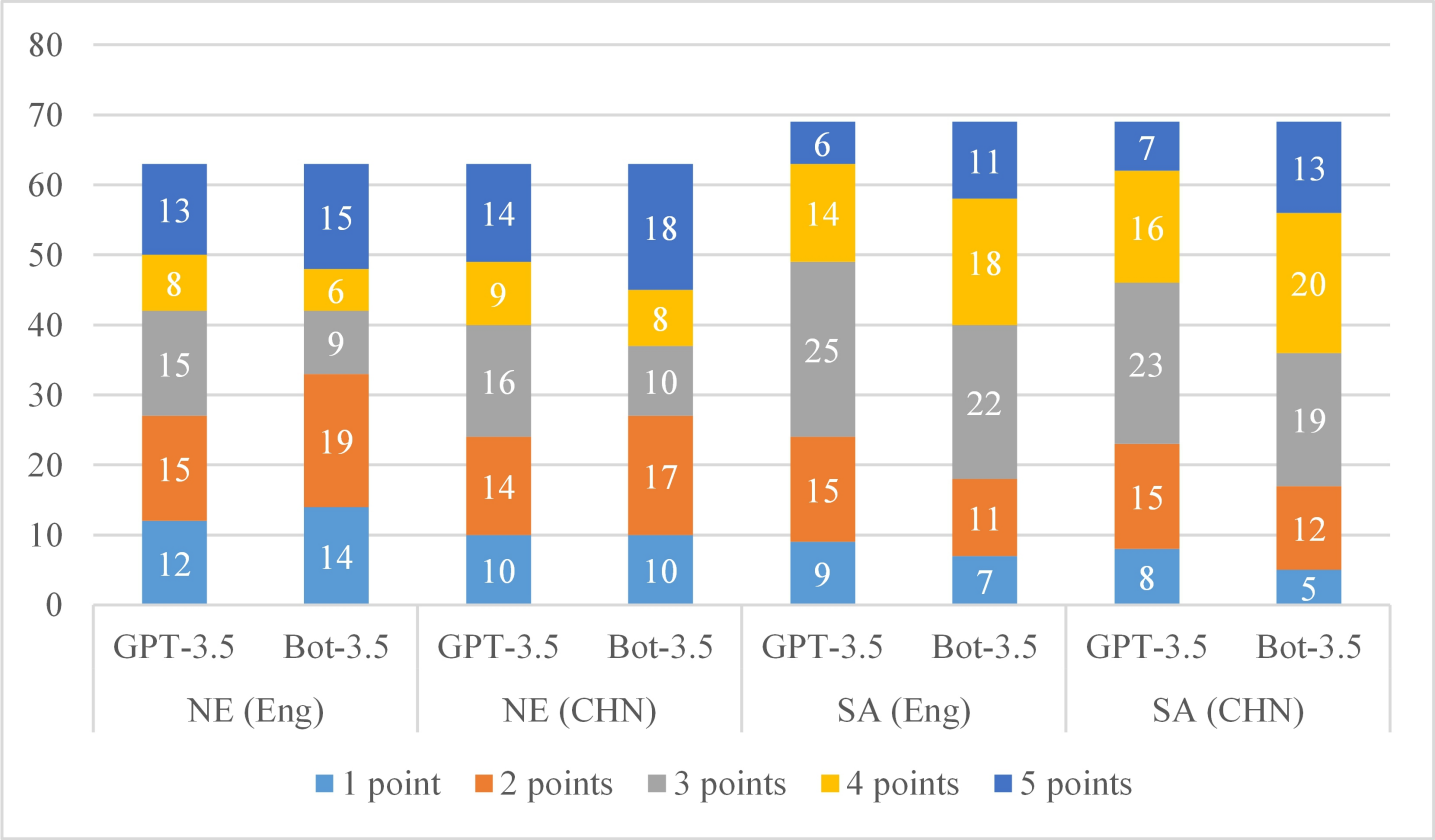
The distribution of scores. Bot-3.5: ERNIE Bot-3.5; CHN: Chinese; Eng: English; GPT-3.5: GPT-3.5 Turbo; NE: noun explanation; SA: short answer.

We collected the scores of the GPT-3.5 and Bot-3.5 AI models for noun interpretation and short answers in both Chinese and English. A total of 63 noun explanation questions and 69 short answer questions were included in this cross-sectional study. The scoring criteria were divided into 5 levels—1 point=very poor, 2 points=poor, 3 points=acceptable, 4 points=good, and 5 points=very good. We take the average score of 5 experts, round the average score to the nearest whole number, and finally calculate classification statistics according to the score values. When asked questions in Chinese (the original language of the test paper), the most common scores were as follows (GPT-3.5 vs Bot-3.5): noun explanation (3 points vs 5 points) and short answer (3 points vs 4 points). When the test paper was translated into English for questions, the most points were scored (GPT-3.5 vs Bot-3.5): noun explanation (2 points vs 2 points) and short answer (3 points vs 4 points). The detailed frequency tables are shown in Table 3 and Figure 2.

**Table 3.** Comparison of the distribution of scores stratified by question type (no explanation, short answer), language (English and Chinese), and artificial intelligence model (GPT-3.5 Turbo [GPT-3.5] and ERNIE Bot-3.5 [Bot-3.5]).

Categories and model		1 point	2 points	3 points	4 points	5 points	Total
**Noun explanation (English)**							
	GPT-3.5	12	15	15	8	13	63
	Bot-3.5	14	19	9	6	15	63
**Noun explanation (Chinese)**							
	GPT-3.5	10	14	16	9	14	63
	Bot-3.5	10	17	10	8	18	63
**Short answer (English)**							
	GPT-3.5	9	15	25	14	6	69
	Bot-3.5	7	11	22	18	11	69
**Short answer (Chinese)**							
	GPT-3.5	8	15	23	16	7	69
	Bot-3.5	5	12	19	20	13	69

We conducted a quantitative statistical analysis of noun explanations and short answer scores, including minimum, maximum, IQR, median, mean, sum, and SD scores. We compared the quantitative statistics of the GPT-3.5 and Bot-3.5 scores in the Chinese language environment. For noun interpretation, the maximum, minimum, and median values were equal for GPT-3.5 versus Bot-3.5; other statistics were as follows: IQR 2‐4 vs 2‐5, mean 2.86 vs 3.37, sum 180 vs 212, and SD 1.4 vs 1.37. There was a significant difference between the 2 models in terms of the quality of answers to noun interpretation (*P*<.05). For short answers, the maximum, minimum, and median values were equal (IQR 2‐4 vs 2.5‐4, mean 2.93 vs 3.36, sum 202 vs 232, and SD 1.15 vs 1.19), and there was a significant difference between the 2 models in terms of the quality of answers to short answers (*P*<.05). The statistical results are shown in Table 4.

**Table 4.** Quantitative statistical results of noun interpretation and short answer scores for 2 artificial intelligence models (GPT-3.5 Turbo [GPT-3.5] and ERNIE Bot-3.5 [Bot-3.5]).

Categories	Noun explanations (n=63)		Short answers (n=69)	
	GPT-3.5	Bot-3.5	GPT-3.5	Bot-3.5
Minimum	1	1	1	1
Maximum	5	5	5	5
IQR	2‐4	2‐5	2‐4	2.5‐4
Median	3	3	3	3
Mean	2.86	3.37	2.93	3.36
Sum	180	212	202	232
SD	1.40	1.37	1.15	1.19

To compare and analyze the performance of the 2 AI models (GPT-3.5 and Bot-3.5) in the Chinese language environment when noun explanations and short answers are classified according to different topics, we calculated the number of scores for each type of topic in the Chinese language environment. For noun interpretation, the highest percentage scores were obtained for GPT-3.5 vs Bot-3.5, basic knowledge (4 points vs 4 points), ultrasound examinations (5 points vs 5 points), disease and etiology (1 point vs 2 points), and ultrasound signs (1 point vs 1 point). For short answers, those with the highest percentage of points (3.5 points vs Bot-3.5), basic knowledge (3 points vs 5 points), ultrasound examinations (2 points vs 3 points), ultrasound diagnosis (3 points vs 5 points), disease and etiology (3 points vs 2 points), and ultrasound signs (3 points vs 3 points) were included. The statistical results are shown in Table 5.

Table 5 also shows the differences in scores between the 2 models on different subject categories. Based on the above results, the most important finding is that Bot-3.5 performs better than GPT-3.5 in the Chinese environment, both objectively and subjectively. Bot-3.5 is representative of the localization model, and GPT-3.5 is representative of the internationalization model. The excellent performance of Bot-3.5 is due to its comprehensiveness and depth in the Chinese training data set. The discovery of this performance difference has implications for both users and developers of chatbot systems, and it is necessary for users to have a deeper understanding of the model’s data set background, language, and performance to better use them. Developers can also improve and optimize robot models according to performance differences.

**Table 5.** The score distributions of noun interpretations and short answers in different topic categories for 2 artificial intelligence models (GPT-3.5 Turbo [GPT-3.5] and ERNIE Bot-3.5 [Bot-3.5]).

Categories	Noun explanations			Short answers		
	Total	GPT-3.5	Bot-3.5	Total	GPT-3.5	Bot-3.5
						
**Basic knowledge**	21			13		
1 point		5	3		3	1
2 points		4	0		1	1
3 points		4	4		4	2
4 points		5	8		2	4
5 points		3	6		3	5
**Examinations**	8			10		
1 point		0	1		2	0
2 points		2	1		4	3
3 points		1	1		1	3
4 points		2	1		2	3
5 points		3	4		1	1
**Diagnosis**	3			10		
1 point		0	1		1	2
2 points		1	0		2	1
3 points		1	1		5	2
4 points		0	0		2	2
5 points		1	1		0	3
**Disease and etiology**	24			7		
1 point		6	0		0	0
2 points		5	9		2	3
3 points		6	6		3	1
4 points		6	3		1	2
5 points		1	6		1	1
**Ultrasound signs**	7			29		
1 point		3	2		2	2
2 points		2	2		8	4
3 points		0	1		10	10
4 points		0	1		7	10
5 points		2	1		2	3

## Discussion

### Preliminary Findings

Since the public release of ChatGPT, its convenience has made AI more accessible than ever before. It has demonstrated its ability to provide answers even to knowledgeable and experienced professors, and its numerous advantages have quickly made it a hot topic and the focus of research. Many technology companies around the world are also developing chatbot systems [4,29,30]. Industries are also exploring how to integrate these AI technologies with their own work and learning to improve quality and efficiency [31,32]. In the medical field, chatbot systems can be used as learning and consulting assistants to answer a variety of medical-related questions, but the accuracy and quality of their information must be carefully evaluated [33,34].

In this cross-sectional study, we analyzed 554 actual examination paper questions from the field of ultrasound medicine and evaluated the performance of an AI chatbot system in answering these medical paper questions. Medical examination papers are often used to evaluate chatbot performance, mainly because of their wide coverage, representativeness, and reference answers [35-37]. Alessandri et al [35] used the Residency Admission National Examination to evaluate ChatGPT. Humar et al [22] evaluated ChatGPT with questions from the plastic surgery in-service examination. The main advantage of our study is that the questions are from actual ultrasound medical examinations, and its content is broad and representative. The question types included single-choice, multiple-choice, true or false questions; noun explanations; short answers; basic knowledge; ultrasound examination; ultrasound diagnosis; ultrasound case analysis; disease and etiology; and ultrasound signs. We conducted a quantitative comparative analysis of the performance of two of the most representative free chatbots (GPT-3.5 and Bot-3.5). Samaan et al [16] demonstrated that chatbot performance is affected by the language environment. We use both English and Chinese to ask questions and analyze the quality of the answers provided by the 2 models in different linguistic environments.

Regardless of whether the input language is English or Chinese, the GPT-3.5 and Bot-3.5 models perform somewhat well on different types of questions in the field of ultrasound medicine. For objective questions (including single-choice, multipl-choice, and true or false questions in this cross-sectional study), we took the accuracy rate as the evaluation index, and a score of ≥60% is acceptable, which is also the passing score of medical students. The best performance was true or false questions (accuracy rate: 60%-80%), followed by single-choice questions (accuracy rate: 57.34%-62.99%), and the worst performance was multiple-choice questions (accuracy rate: 8.33%-39.58%). For subjective questions (including noun explanations and short answers in this cross-sectional study ≥3 points, considered to be acceptable answers, also the assessment criteria), the performance was better for short answers, with acceptable answers (≥3 points) accounting for 65.22%-75.36%, whereas the performance for noun explanations was lower, with acceptable answers accounting for 47.62%-61.9%.

The reasons behind these differences are worth exploring and analyzing. For objective questions, the difficulty and complexity of the questions may be important factors. Branum and Schiavenato [14] reported that chatbots sometimes provide plausible but incorrect answers to complex clinical questions [38]. True or false questions usually involve only the truth or falsity of a statement and are the least difficult. Although multiple options are provided in a single choice, the answer is unique, and the model needs to identify only the correct answer from a limited number of options, with moderate difficulty. The research results of Lai et al [32] indicate that chatbots have high accuracy in single-choice selection. However, multiple choice requires the model to identify the correctness of multiple options at the same time, which significantly increases the difficulty and complexity of information processing. Mihalache et al [3] demonstrated that substantial progress has not been made in multiple-choice chatbots. Furthermore, the processing of multiple-choice questions in the process of model training may be less common than that of other types of questions, and most of the multiple-choice answers are more flexible. For subjective questions, differences in openness, flexibility, and scoring criteria may be the reasons for differences in performance. Short answer questions often require the model to provide a short paragraph of explanation or description, which allows the model to show more flexibility and depth in its responses. On the other hand, noun interpretation is more focused on the precise interpretation of specific terms, which requires higher accuracy and professionalism of the model. When short answers are graded, the grading criteria may be more flexible, allowing a degree of freedom. Other studies have asked questions about diseases similar to the short-answer questions in this study. Yun et al [33] demonstrated the effectiveness of chatbots when short-answer questions are answered. Noun interpretation, on the other hand, can be more rigorous, requiring the model to provide a precise and professional interpretation [39]. Therefore, the differences in the performance of the models on different question types can be attributed to the differences in the difficulty of the questions, the complexity of information processing, the training data, and the scoring criteria. As a user, it is necessary to understand the performance differences of models of different types in advance, and as a developer, adjusting the distribution of the training data set and optimizing the model are important.

When comparing the performance of the GPT-3.5 and Bot-3.5 AI models in the field of ultrasound medicine, we found some significant differences. Table 2 reported that the accuracy rate of GPT-3.5 for multiple-choice and true or false questions is lower than that of Bot-3.5, suggesting that Bot-3.5 may be more capable of handling problems that require deep understanding and reasoning. Tang and Yang [23] demonstrated that Bot-3.5 shows greater participation and enthusiasm in teaching applications. This difference may be related to the differences in training data and algorithm architecture between the 2 models. Bot-3.5 may have been exposed to more diverse and complex data from the medical field during training to be better able to handle these types of problems. From Tables 4 and 5, we further analyze the scores of the 2 models on different question types. In terms of noun interpretation and short answers, Bot-3.5 scores are generally higher than GPT-3.5 scores, especially in the Chinese context. This result suggested that Bot-3.5 may have greater accuracy in understanding and interpreting medical terms and concepts. In addition, from the comparison of the score statistics, we can observe that Bot-3.5 is more concentrated in the score distribution, which implies that it has greater stability and consistency in dealing with similar problems.

When the test paper was translated into English for questioning, the performance of both models declined, but Bot-3.5 maintained its advantage in terms of multiple-choice and true or false questions. This finding reveals the limitations of both models in cross-language processing. Zhu et al [25] compared the performance of large language models developed in different countries, highlighting the necessity for localized models. Although both models claim to support multilingual processing, in practice, the model’s ability to process across languages is affected by various aspects, such as architecture, algorithms, or training data, and when the model switches from 1 language to another, it may need to readjust to new language features. This adaptation process can lead to performance degradation, especially when dealing with complex tasks. During the training of the model, there may be differences in the distribution and scale of the Chinese and English data, and if the model is trained more fully on the Chinese data, it may perform better on the Chinese input. Bot-3.5 was developed and trained in China and has a large Chinese corpus. During the design and training process, Chinese language features were deeply optimized. This optimization allows Bot-3.5 to perform well in terms of Chinese semantic understanding and context grasp.

Biswas et al [26] conducted a categorical assessment analysis of these problems. In terms of noun interpretation and short answer questions, both models scored highly in basic knowledge, ultrasonography, disease, and cause, which may be related to the importance of these topics in the medical field and their richness in the training data. However, in areas such as ultrasound signs and ultrasound diagnosis, both models scored relatively low, which may reflect the complexity and challenge of these topics in the medical field, requiring more in-depth reasoning and understanding of complex issues [40].

Although chatbots are not yet able to provide perfect answers in all aspects of medicine, chatbots have great potential for answering medical questions. Because the performance of chatbots is not static, with the continuous enrichment of training data and the continuous improvement and optimization of algorithms, chatbot performance will continue to improve. The results of this cross-sectional study have implications for both users and developers of chatbot systems. To improve the quality and efficiency of chatbot use, users need to deeply understand the performance and characteristics of different models and carefully study the performance differences and evaluation results of different models on different topics or task types to select the appropriate model according to actual needs [24]. Moreover, users should set clear expectations and establish appropriate evaluation criteria based on the model's actual performance to conduct objective and comprehensive evaluations in practical applications. For complex tasks or tasks requiring high accuracy, users can attempt to combine the advantages of multiple models, adopt the method of ensemble learning, and weight the prediction results of multiple models to obtain more accurate prediction results. Specific feedback and suggestions are provided to developers in appropriate ways and channels when the model is used. For developers, the results of the study can be used to optimize the model for performance issues on a particular topic or task. For example, training data in related fields can be increased, or more advanced training algorithms can be used to improve the performance of the model in the medical field. The user feedback and suggestions are actively collected, and the model is constantly iterated and improved. Everyone needs to recognize that no chatbot is omnipotent. We need to understand it, use it correctly rather than contradict or abuse it, treat it as a judge, let it grow healthily, and create more and better value for human beings.

### Limitations

Our study has several limitations. First, the original language of all the questions in this cross-sectional study was Chinese, and the questions were asked in Chinese or translated from Chinese to English, which may not represent the performance of the model in other languages. Second, the sample size of our study was not large and focused only on text-related topics related to ultrasound medicine. Experts also have potential bias in the scoring. The universality of the results of this study in other medical specialties needs further research. Finally, chatbot versions are constantly being updated, and the results of this study may not be representative of the performance of the most recent version of the model at the time of publication [41]. However, GPT-3.5 and Bot-3.5 are the best-performing versions of the current free versions, as well as the largest number of users, and it is meaningful to study the performance comparison of the latest free versions of each model over the same period.

### Conclusions

The results show that the AI chatbots represented by GPT-3.5 have a certain ability to answer questions in ultrasound medical examination papers, but there are varying degrees of performance differences across chatbot models, input languages, question types. and topics. For the Chinese ultrasound medical questions in this study, Bot-3.5 was superior to GPT-3.5 in terms of both accuracy and quality in many aspects. These findings suggest that users need to thoroughly understand the performance characteristics of various models and their applicability to different types of problems. For complex problems, multiple models are needed for comprehensive analysis. This finding also suggests that developers need to continuously optimize models to enrich training data, especially in medical specialties. In this way, chatbots can be continuously optimized, their performance consistently improved, and their ability to provide high-quality services enhanced.

## Abbreviations

AI: artificial intelligence
Bot-3.5: ERNIE Bot-3.5
GPT-3.5: GPT-3.5 Turbo
